# Removal of fluoride from water using a novel sorbent lanthanum-impregnated bauxite

**DOI:** 10.1186/s40064-016-3112-6

**Published:** 2016-08-26

**Authors:** C. M. Vivek Vardhan, M. Srimurali

**Affiliations:** Department of Civil Engineering, Sri Venkateswara University, Tirupati, Andhra Pradesh 517501 India

**Keywords:** Fluoride, Water, Removal, Adsorption, Lanthanum, Bauxite

## Abstract

A novel sorbent, Lanthanum-Impregnated Bauxite (LIB), was prepared to remove fluoride from water. To understand the surface chemical composition and morphology, LIB was characterized using X-ray diffraction and scanning electron microscopy techniques. Experiments were performed to evaluate the sorption potential, dose of sorbent, kinetics, equilibrium sorption capacity, pH and influence of anions for defluoridation by LIB. Equilibrium isothermal studies were conducted to model the sorption and regeneration studies were carried out to evaluate the reusability of LIB. The results showed that LIB, at a dose of 2 g/L could remove 99 % of fluoride from an initial concentration of 20 mgF/L. Kinetic studies revealed the best fit of pseudo second order model. The sorption followed Langmuir isotherm model and the maximum sorption capacity of LIB for removal of fluoride was found to be 18.18 mg/g. Naturally occurring pH of water was found to be favorable for sorption. Usually occurring anions in water except nitrates influenced sorption of fluoride by LIB.

## Background

Excessive fluoride in drinking water causes serious health problems such as brittleness of bones, dwarfishness, fluorosis and cancers (Chinoy 1991). The maximum contaminant level (MCL) of fluoride in drinking water is 1.5 mg/L, according to the World Health Organization (2004). Groundwater with fluoride concentration >1.5 mg/L is prevalent in several regions of the world, warranting treatment (Yeşilnacar et al. 2016; Atasoy et al. 2013; Vijaya Kumar et al. 1991; Gaciri and Davies 1993; Czarnowski et al. 1996). Several technologies such as adsorption (Vivek Vardhan and Karthikeyan 2011), coagulation and flocculation (Emamjomeh and Sivakumar 2006), electrodialysis (Adhikary et al. 1989), electrocoagulation (Khatibikamala et al. 2010) and reverse osmosis (Simons 1993) have been tried to remove fluoride from water with varying degrees of success. Chemical precipitation of fluoride using alum and lime, known as Nalgonda Technique (Nawlakhe et al. 1978) can be used for fluoride removal. However, it poses some problems such as generation of large volumes of sludge, which is difficult to deal with. Adsorption is considered to be a feasible technique especially for household applications or for small communities (Srimurali et al. 1998). Various sorbents such as activated alumina (Boruff 1934; Fink and Lindsay 1936; Swope and Hess 1937), bone char (Nemade et al. 2002), bauxite (Sujana and Anand 2011), magnesium amended activated alumina (Maliyekkal et al. 2008) and rice husk (Vivek Vardhan and Karthikeyan 2011) have been tried (Bhatnagar et al. 2011; Ayoob et al. 2008). Among various adsorbents used activated alumina is deemed to be the selective sorbent for removal of fluoride from water (Boruff 1934; Fink and Lindsay 1936; Swope and Hess 1937). However, due to some drawbacks such as optimum removal at a low pH value of 5.5, its practical scope of applicability is limited.

Recently various rare earth materials such as lanthanum (Na and Park 2010), lanthanum modified activated alumina (Cheng et al. 2014), lanthanum oxide (Nagendra Rao and Karthikeyan 2012), lanthanum impregnated green sand (Vivek Vardhan and Srimurali 2016), cerium (Xu et al. 2001) and yttrium (Raichur and Basu 2001) have been used as sorbents for removal of fluoride from water. Though lanthanum has got good affinity for fluoride, there are some difficulties related to its use as an adsorbent. Compounds of lanthanum are present in fine powder form. Application of lanthanum compounds in powder form for adsorption is associated with practical limitations such as difficulty in separation from liquid, impeded hydraulic flow and leachate of metal with treated water (Maliyekkal et al. 2008). To overcome these problems, lanthanum had to be fixed onto a suitable substrate. Bauxite is an ore of aluminum and is abundantly available at low cost. In the present investigation, an attempt has been made to impregnate lanthanum onto bauxite, in order to develop a low-cost adsorbent and also to study the synergetic effect of lanthanum and bauxite on fluoride removal as well as to overcome the drawbacks associated with the use of lanthanum powder. Lanthanum Impregnated Bauxite (LIB) was prepared using La_2_CO_3_. La_2_CO_3_ is the base material for synthesis of other forms of Lanthanum and is available at low-cost. Also the quantity of La_2_CO_3_ that goes into impregnation for synthesis of LIB is very less. So, when used on a massive scale, LIB turns out to be a very low-cost adsorbent. However, the exact cost analysis will be done in future studies. LIB was characterized using X-ray diffraction (XRD) studies and Scanning Election Microscopy (SEM). Adsorption experiments were conducted in batch mode. Experiments involving Kinetics, isothermal equilibrium, pH and regeneration studies were carried out to evaluate the practical feasibility of application of LIB as an adsorbent for removal of fluoride from water.

## Methods

### Chemicals

All reagents used in the present investigation were of analytical grade and procured from E. Merck Ltd, India. Water used in all batch sorption studies was laboratory distilled water prepared with a glass distillation unit (pH 6.7 ± 0.1 and specific conductivity 2.0 to 4.3 µS/cm). Stock solution of fluoride of 100 mg/L was prepared with distilled water using sodium fluoride. Aqueous fluoride solution was prepared by adding appropriate quantity of stock fluoride solution into distilled water and used in all adsorption experiments unless otherwise specified. LIB was prepared by thermal impregnation method as described below in adsorbent preparation. Raw bauxite was collected from mines at Mahboobabad, India. Lanthanum carbonate was purchased from Indian Rare Earths Limited, Aluva, Kerala, India.

### Adsorbent preparation

Raw bauxite was crushed and sieved to get <75 micron particle size. Bauxite so obtained was heated in a muffle furnace at 400 °C for 4 h. This heated bauxite was cooled to room temperature in a desiccator and is called calcined bauxite. Calcined bauxite was stored in an air tight plastic container for further use. In a separate conical flask, La_2_(CO_3_)_3_ of 0.5 g was mixed with 50 mL of distilled water and dilute HCl was added to it drop wise till the La_2_(CO_3_)_3_ got completely dissolved. To this solution 20 g of prepared calcined bauxite was added and mixed using a magnetic stirrer for 3 h. The liquids were strained off and the solid material obtained was washed with distilled water. It was dried in a water bath at 110 °C for 6 h and subsequently heated in a muffle furnace at 950 °C for 4 h and then cooled. The material thus obtained was called LIB and used as an adsorbent in all further investigations. LIB was processed at high temperatures for lanthanum impregnation, whereas bauxite was calcined to improve its surface properties. So, this paper has the limitation of not studying bauxite and LIB, subject to same thermal treatment to bring out the exact differences due to lanthanum impregnation. Calcined bauxite is hereafter referred to as simply bauxite in this paper.

### Characterization of adsorbent

LIB samples were analyzed by X-ray powder diffraction (XRD) technique before and after adsorption for studying its mineralogy. XRD analysis was carried out using a X-ray diffractometer, Philips: PW1830 with CuKα radiation. To study the surface morphology, scanning electron microscope (SEM) was used. SEM and EDAX images were obtained from a Carl Zeiss, EVO MA15 instrument. Particle size distribution was analyzed using Ankersmid particle size analyzer. Pore size analysis of bauxite and LIB were done by using a micropore analyzer (ASAP 2020, Micromeritics, USA) by Nitrogen chemisorption isotherm technique (Carabineiro et al. 2011).

### Batch adsorption experiments

Sorption experiments were conducted in batch mode using 250 mL Teflon flasks with a 100 mL of 20 mg/L of aqueous fluoride solution. A known quantity of adsorbent was added to the prepared fluoride solution in Teflon flasks. It was agitated using a rotary shaker of make Kaizen Imperial at 160 rpm and at room temperature for specific contact periods ranging from 0 to 360 ± 1 min. The solutions contained in the flasks were then withdrawn at specified contact periods, filtered with 42 Whatman filter paper of pore size 2.5 µm and analysed for residual fluoride using SPADNS method (APHA) (APHA et al. 1996) at 570 Nm. A spectrophotometer, Evolution 201, of Thermo Scientific make was used to analyze fluoride. The contact period, at which there was no further reduction of fluoride, is considered the equilibrium contact time. Similarly the optimum usage of adsorbent was studied by varying the sorbent dose ranging from 0 to 8 ± 0.01 g/L for a constant equilibrium contact time. To understand the influence of pH, sorption experiments were conducted at different pH values ranging from 2 to 12. Starting pH adjustments were made using diluted NaOH and H_2_SO_4_. pH was measured using a Hanna make, pH analyzer. Optimum values obtained during preliminary investigations for various parameters were used in all further detailed experimentation. Fluoride ion concentrations varying from 5 to 70 mg/L were used in sorption equilibrium investigations, to arrive at the best fitting isothermal model. The reporting fluoride concentration range by SPADNS method is from 0 to 1.4 ± 0.1 mg/L. Appropriate dilutions of samples were made when fluoride exceeded the above mentioned concentration range. Concentrations of lanthanum and aluminum were measured using atomic absorption spectrometer with a graphite furnace (AAS, GBC 932 Plus).

### Kinetics of sorption

In the present investigation pseudo first order, pseudo second order and intraparticle diffusion models were studied to understand the kinetics of adsorption of fluoride using bauxite and LIB.

#### Pseudo first order equation and pseudo second order equation

 The mathematical equation of pseudo first order equation is as given in Eq. (1) (Lagergren 1898).

1 $${\text{Log}}\left( {{\text{q}}_{\text{e}} - {\text{q}}_{\text{t}} } \right) = \left( {{\text{Log}}\;{\text{q}}_{\text{e}} } \right) - \left( {{\text{K}}_{1} {\text{t/}}2.303} \right)$$

where q_e_ represents adsorbed fluoride at equilibrium and q_t_ represents adsorbed fluoride at time t·k_1_ (L/min) represents rate constant of adsorption. A plot was drawn between (t) versus Log (q_e_ − q_t_). K_1_ and q_e_ were obtained from its slope and intercept. The linear form of mathematical equation for pseudo second order model is given in Eq. (2) (Ho and McKay 1999).

2 $${\text{t/q}}_{\text{t}} = \left( {1 / {\text{K}}_{2} {\text{q}}_{\text{e}}^{2} } \right) + \left( {{\text{t/q}}_{\text{e}} } \right)$$

where, K_2_ is a rate constant.

#### Intraparticle diffusion analysis

To design and control an adsorption system the mechanism involved and the rate limiting step are to be determined. In a well agitated system, migration of sorbate from a bulk solution to surround the adsorbent is not difficult (Weber and Morris 1963). Therefore bulk transport is rapid and it cannot be rate limiting. Similarly sorption of fluoride ions onto the active sites of sorbent is rapid and so this too cannot be a rate limiting step (Na and Park 2010). So, either film diffusion or intraparticle diffusion acts as rate slowing step or eventually becomes the rate controlling step (Yousef et al. 2011). To identify the rate controlling step and also to understand the mechanism involved in sorption, intraparticle diffusion equation, derived from unsteady state diffusion in flat plate is employed, which is given in Eq. (3) (Oliveira et al. 2005)

3 $${\text{q}}_{\text{t}} = {\text{k}}_{\text{id}} \cdot {\text{t}}^{1/2} + {\text{C}}$$

where, C is a constant, proportional to boundary layer thickness and k_id_ is rate constant. If the plot of q_t_ versus t^1/2^ is linear, it indicates the involvement of intraparticle diffusion. If the obtained straight line passes through, origin then it indicates that intraparticle diffusion alone is the rate limiting step. Conversely if the obtained straight line does not pass through origin, it indicates involvement of some other mechanism, in addition to intraparticle diffusion (Oliveira et al. 2005).

### Effect of competing ions

The influence of anions on the efficiency of fluoride sorption by LIB was investigated. In order to find this, various individual ions of Cl^−^, SO_4_^2−^, PO_4_^3−^, HCO_3_^−^ and NO_3_^−^ of concentrations up to 100 mg/L were added each separately into 20 mg/L of aqueous fluoride solution and adsorption experiments were carried out using 2 g/L of LIB as well as 6 g/L of bauxite. The liquid samples were then withdrawn after reaching equilibrium time and analyzed for residual fluoride concentrations. Wherever the influence of phosphates was analyzed, for every 16 mg/L of PO_4_^3−^, an error correction of −0.1 mg/L was made to rectify its interference with SPADNS method (Hach company 1989–2014).

### Determination of pH zero point charge

pH of zero point charge (pH_zpc_) was found by a batch equilibrium method (Rivera-Utrilla et al. 2001). Typically, NaCl of 0.01 M concentration and 50 mL in quantity was taken into six conical flasks. pH of these solutions were varied between 2 and 12 using H_2_SO_4_ or NaOH. One gram of LIB was added to each flask and a plot was drawn between pH before addition of LIB and pH after addition of LIB. This plot yielded a straight line. The flasks were then agitated at room temperature for 48 h and then the pH values were noted. Now a plot was drawn between pH value of solution before agitation and pH value after 48 h of agitation, which eventually yielded a curve. The intersection point of the straight line and the curve is the value of pH_zpc_ of LIB. Similar experiments were conducted with bauxite. Results obtained by this method are in close agreement with the results obtained by Carabineiro et al. (2011).

### Regeneration experiments

After the agitated batch sorption experiments were conducted under optimal conditions with 20 mg/L aqueous fluoride solution, the liquids were strained off and the sorbent which got loaded to capacity was air dried for 48 h. Further it was desorbed by agitation with various eluents such as distilled water, NaOH and HCl, for a period of 180 min. The best desorbent was considered as the regeneration agent.

### Cycles of regeneration

Regular batch adsorption experiments were conducted with a 20 mg/L of aqueous fluoride solution using adsorbent under optimal experimental conditions. After sorption, the spent sorbent, which got loaded to capacity was separated by filtration using a 42 Whatman filter paper and air dried for 48 h. Subsequently it was desorbed using the most appropriate eluent found through experimentation. Such regenerated sorbent was again separated and air dried to be used as a fresh sorbent for removal of fluoride from a 20 mg/L of aqueous fluoride solution. After agitation, the residual fluoride in solution was measured. This process was repeated several times until the residual fluoride exceeded the permissible limits. The number of cycles until fluoride in solution reached the permissible limit was considered the optimum cycles of reusability of sorbent.

## Results and discussion

### Sorbent characterization

**Fig. 1:**
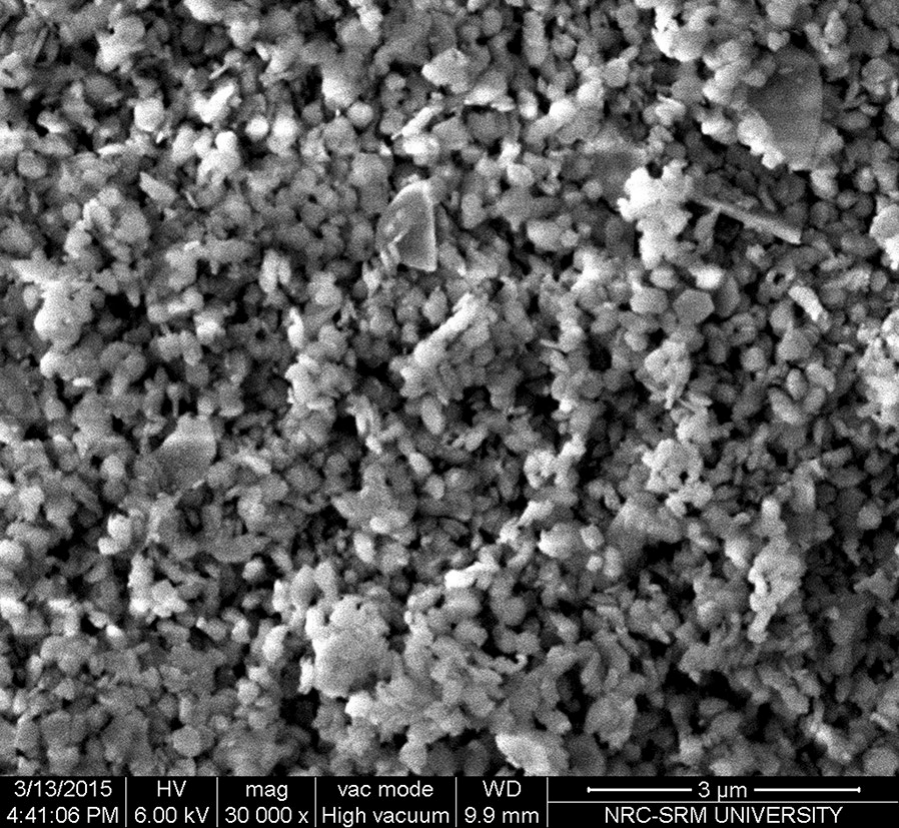
SEM image of LIB

SEM image and EDAX spectra of LIB are presented in Fig. 1 and Table 1 respectively. The white colored dense precipitates observed could be attributed to impregnated lanthanum on the background granules of bauxite. In the EDAX spectrum, the elements, Al, La, Ti and Fe can be noticed, which give indirect evidence of presence of lanthanum on bauxite. From the particle size distribution analysis, it was observed that more than 90 % of LIB and bauxite particles/aggregates fell in the range of 40–55 µm.

**Table 1:** EDAX of LIB

Element	Atomic number	Series	Unn.C (Wt%)	Norm.c (Wt%)	Atom.c (at.%)	Error (1 sigma) (Wt%)
Ti	22	L-series	68.8	64.47	58.48	16.60
Al	13	K-series	21.13	19.8	31.88	1.28
Fe	26	L-series	10.85	10.17	7.91	3.87
La	57	M-series	5.93	5.56	1.74	4.38

Unn.C (Wt%): The unnormalised concentration in weight percent of the element

Norm.C (Wt%): The normalised concentration in weight percent of the element

Atom.c (at.%): The atomic weight percent

Error (1 sigma) (Wt%): The error in the weight percent concentration at the 1 sigma level

### Influence of sorbent dose

**Fig. 2:**
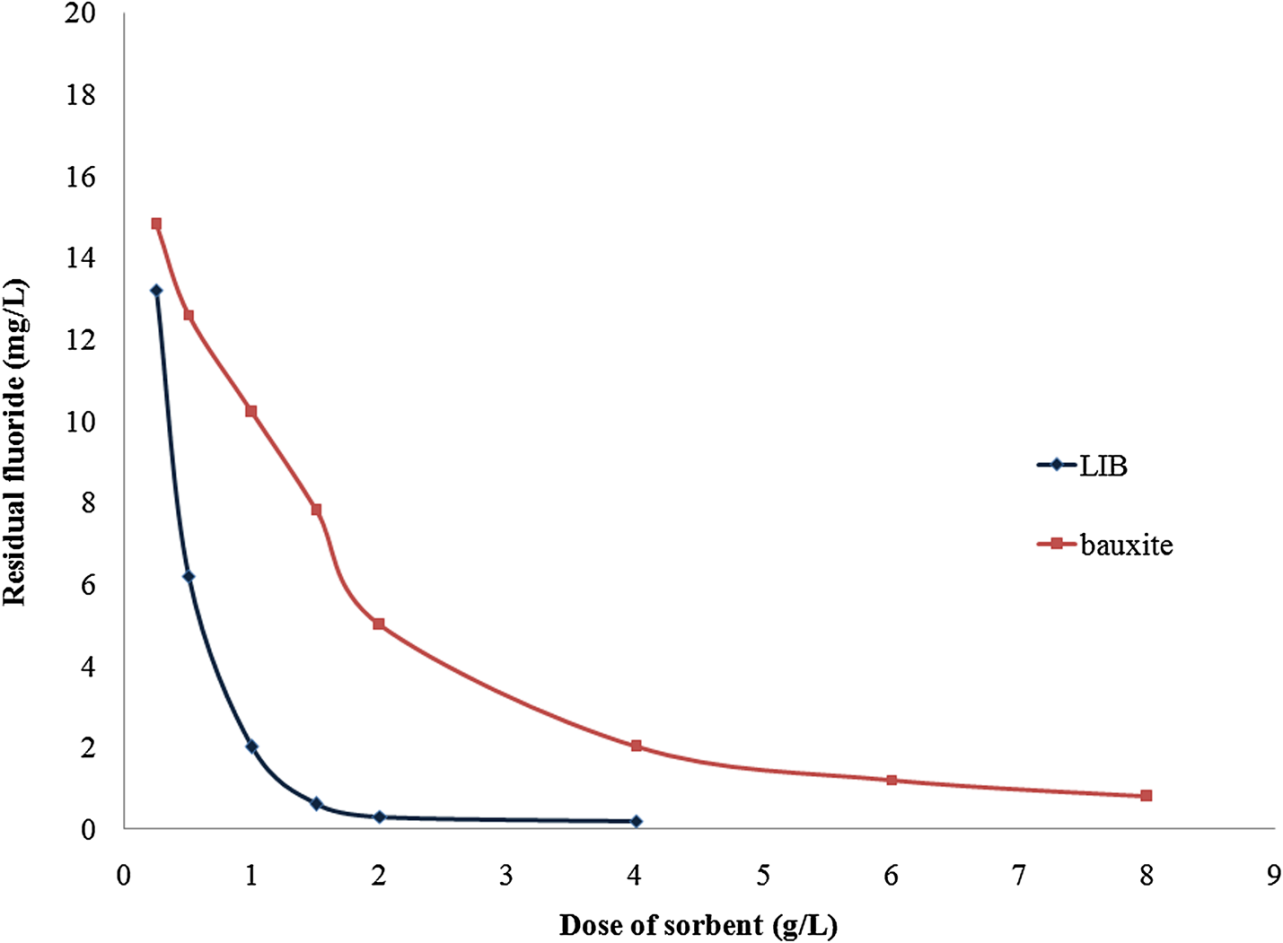
Comparison of influence of doses of LIB and bauxite on fluoride removal. Initial fluoride = 20 mg/L

Experiments were conducted on LIB and bauxite separately to find their dose required for removal of fluoride from water. The corresponding experimental results are presented in Fig. 2. It can be observed from the figure that LIB at a dose of 2 g/L could remove 99 % of fluoride from an initial fluoride concentration of 20 mg/L, whereas bauxite at 6 g/L could remove 94 % of fluoride from an initial fluoride concentration of 20 mg/L. Removal of fluoride by bauxite was low, compared to LIB, possibly due to high affinity of lanthanum for fluoride. The concentration of lanthanum and aluminum in treated water were found to be 0.05 ± 0.01 and 0.02 ± 0.01 mg/L respectively, which are not harmful (Feng et al. 2006; Bureau of Indian Standards 2012).

### Kinetic studies

#### Influence of contact time

**Fig. 3:**
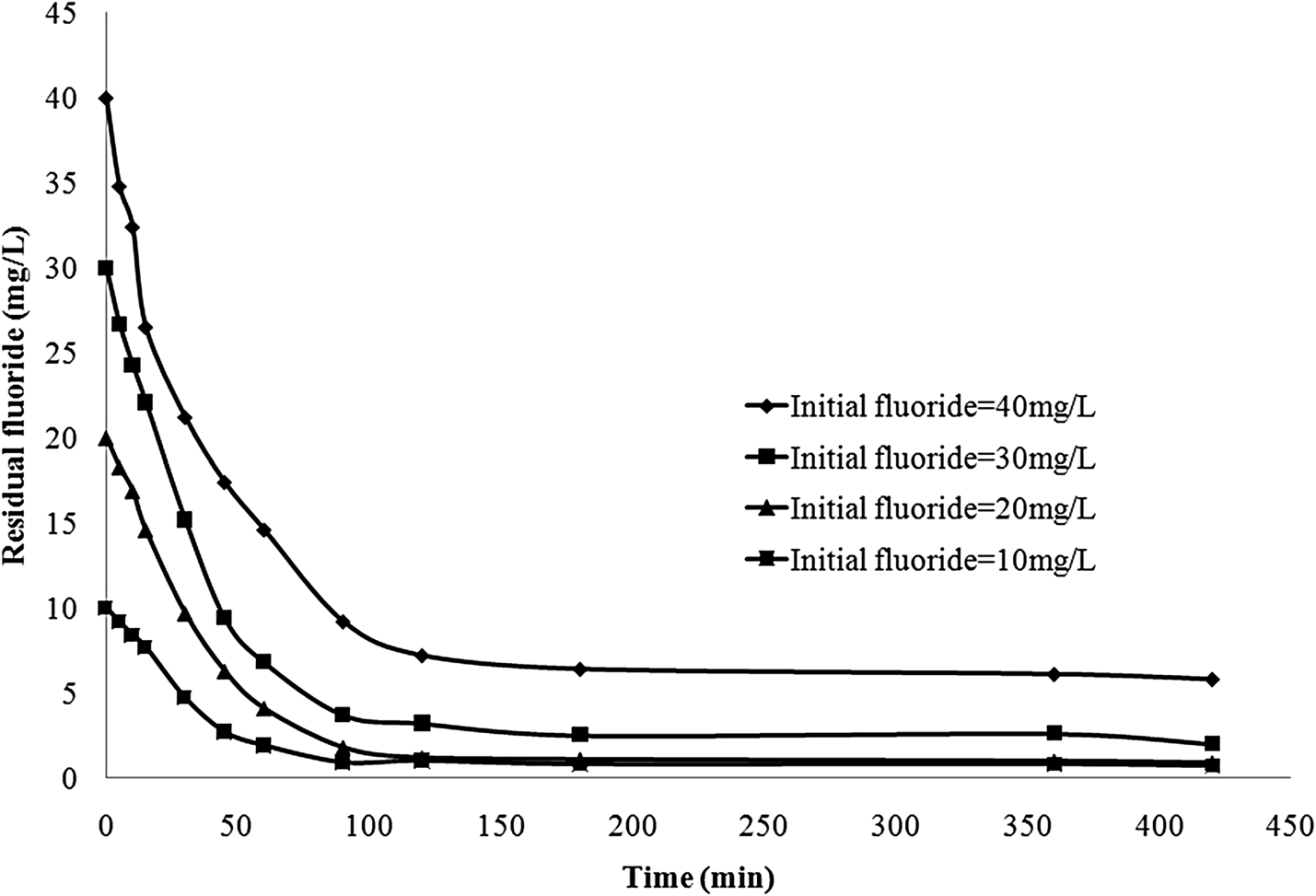
Kinetics of fluoride removal by LIB at various initial concentrations of fluoride (adsorbent dose = 2 g/L)

**Fig. 4:**
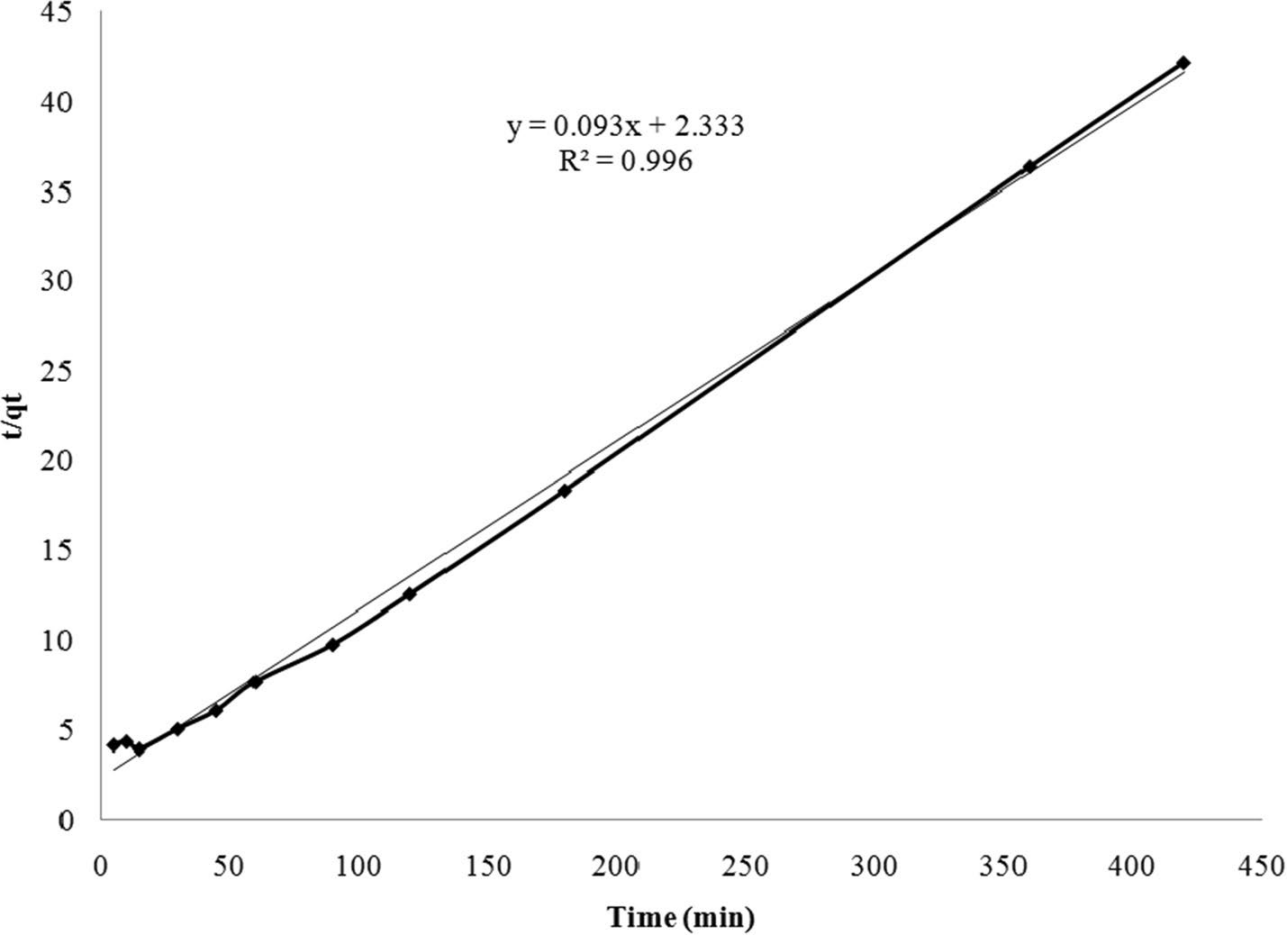
Plot of pseudo-second-order equation for sorption of fluoride onto LIB Adsorbent dose = 2 g/L. Initial fluoride = 20 mg/L

**Fig. 5:**
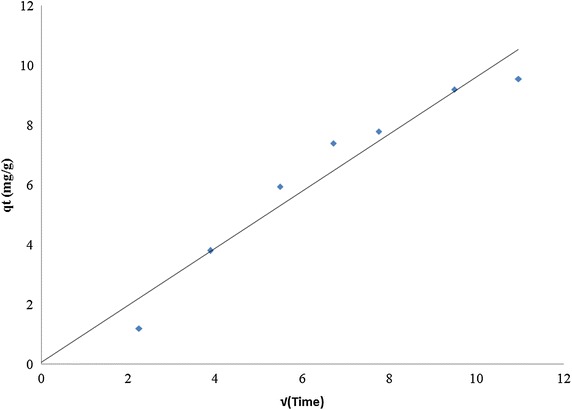
Intraparticle diffusion plot for sorption of fluoride onto LIB

Attaining equilibrium of adsorption during an adsorption process involves various diffusion mechanisms before the sorbate finally adsorbs onto the active adsorption sites on the sorbent (Biswas et al. 2009). Adsorption kinetics explains the rates at which different stages involving various mechanisms proceed. In the present study the time taken for adsorption of fluoride onto bauxite and LIB was investigated. It was observed that it took 120 min for removal of fluoride to below 1.5 mg/L using bauxite (Figure not shown). Figure 3 shows the time taken for sorption of various concentrations of fluoride onto LIB. Sorption was rapid in the initial 30 min. Later the rate of adsorption got stabilized. The plot of pseudo second order model for sorption of fluoride onto LIB is given in Fig. 4. The calculated parameters of the above two models for both bauxite and LIB are presented in Table 2. It can be observed from the obtained R^2^ values, that pseudo second order model fits best to both bauxite and LIB. Figure 5, depicts the plot between q_t_ versus t^1/2^ for LIB. It can be observed from the figure that the plot yielded almost a straight line tending to pass through origin. This suggests that intraparticle diffusion alone is the rate limiting step. In general, in a well agitated system, film diffusion cannot be a rate limiting step (Weber and Morris 1963). It can be observed from Table 2 that Pseudo-second order model fits better to both LIB as well as to bauxite, based on regression analysis. This suggests a predominance of involvement of active chemical sites that aid in the process of sorption. The pore size characteristics of bauxite, LIB and activated alumina (Maliyekkal et al. 2008) are presented in Table 3. It can be observed that the pore size diameter has got reduced from 63 (Bauxite) to 54 nm (LIB), possibly due to lanthanum impregnation. The pores in activated alumina fall in mesoporous range and that of bauxite and LIB fall in macroporous range according to IUPAC classification (Everett 1973, 1976). This explains the high rate of sorption of fluoride onto LIB.

**Table 2:** Comparison of parameters of kinetic models for adsorption of fluoride onto LIB and bauxite

	Pseudo-first-order	Pseudo-second-order	Intraparticle diffusion
LIB	qe (exp) = 9.8 (mg/g) qe (cal) = 5.462 (mg/g) K_1_ = 0.0152 (min^−1^) R^2^ = 0.8921	qe (cal) = 10.75 (mg/g) K_2_ = .0037 (min^−1^) R^2^ = 0.9965	K_id_ = 0.438 (g mg^−1^ min^−1^) C = 2.9041 R^2^ = 0.6986
Bauxite	qe (exp) = 3.1666 (mg/g) qe (cal) = 0.9156 (mg/g) K_1_ = 0.0154 (min^−1^) R^2^ = 0.8956	qe (cal) = 1.781(mg/g) K_2_ = 0.0225 (min^−1^) R^2^ = 0.9964	K_id_ = 0.073 (g mg^−1^ min^−1^) C = 0.4837 R^2^ = 0.699

**Table 3:** Pore size characteristics of bauxite, LIB and activated alumina

Pore characteristics	Bauxite	LIB	Activated alumina
BET surface area	7 m^2^/g	14 m^2^/g	242 m^2^/g
BJH adsorption cumulative volume of pores between 17.000 and 3000.000 Å diameter	0.09 cm^3^/g	0.17 cm^3^/g	0.29 cm^3^/g
BJH Desorption cumulative volume of pores between 17.000 and 3000.000 Å diameter	0.13 cm^3^/g	0.17 cm^3^/g	0.30 cm^3^/g
Adsorption average pore width (4 V/A by BET)	79 nm	49 nm	5 nm
BJH Adsorption average pore diameter (4 V/A)	63 nm	54 nm	5 nm
BJH Desorption average pore diameter (4 V/A)	48 nm	43 nm	5 nm

### Equilibrium isothermal studies

**Fig. 6:**
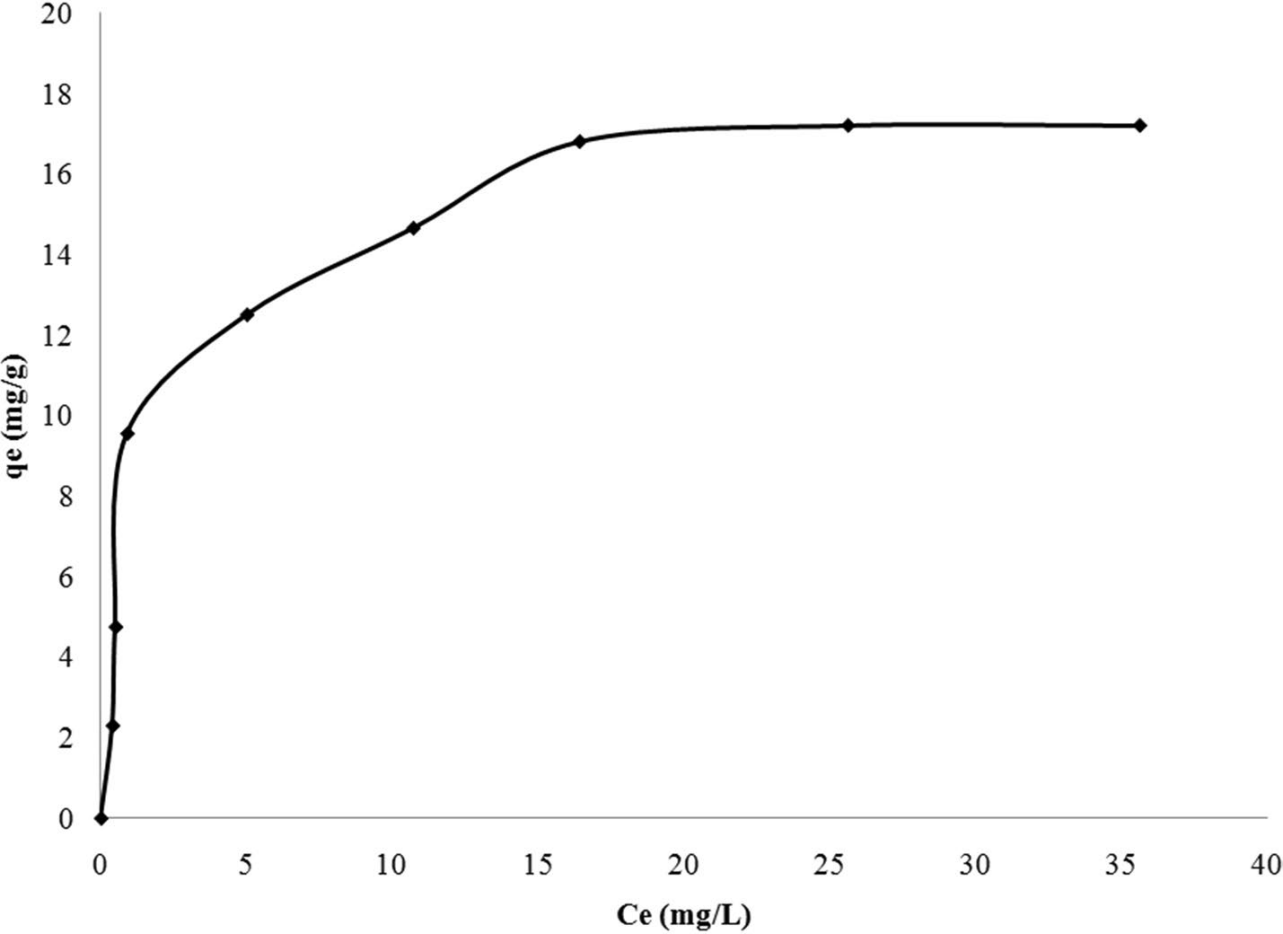
Standard isotherm plot of sorption of fluoride onto LIB (Fluoride concentration from 5 to 70 mg/L)

**Fig. 7:**
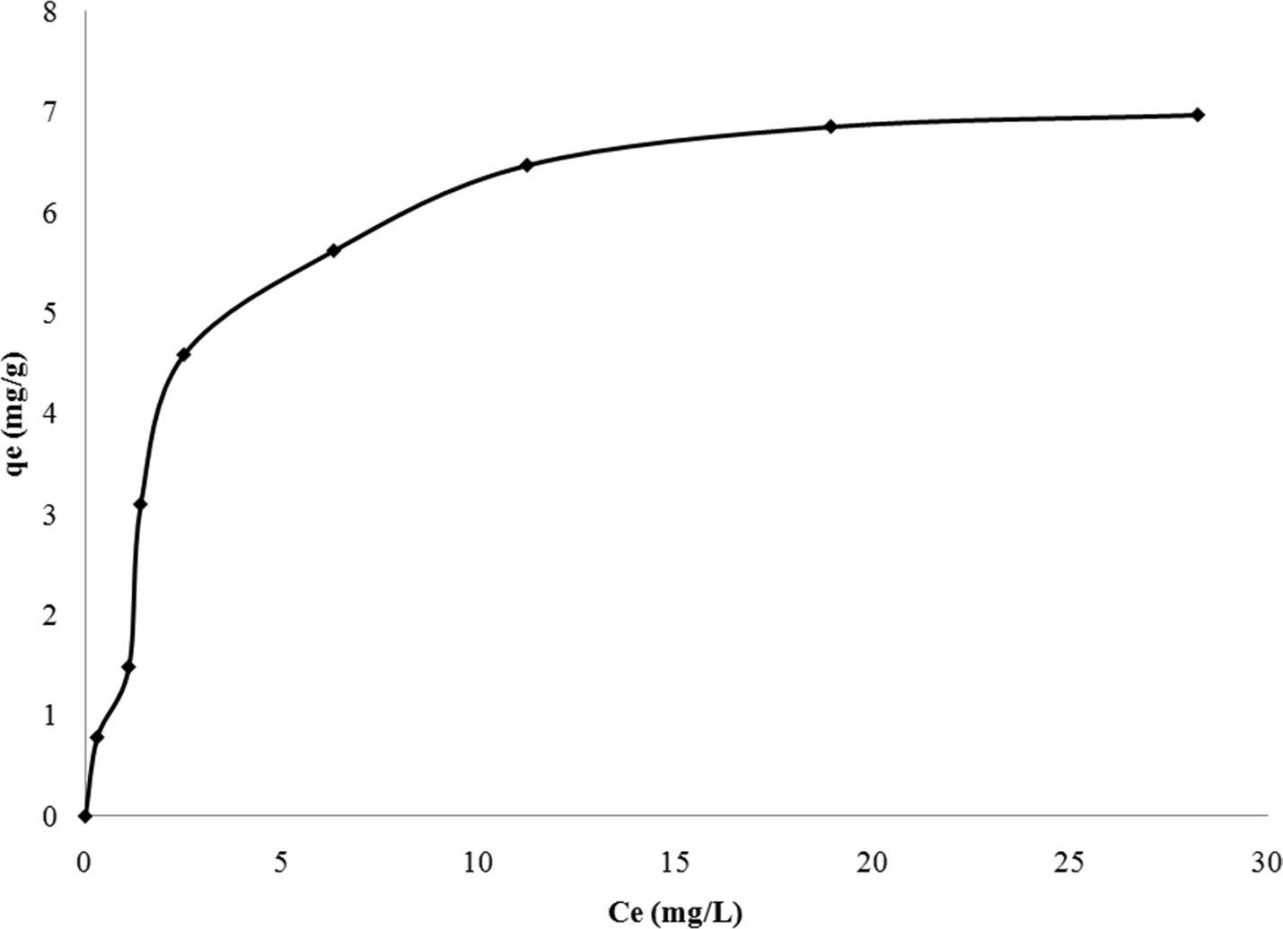
Standard isotherm plot of sorption of fluoride onto bauxite (Fluoride concentration from 5 to 70 mg/L)

Equlibrium isothermal studies are conducted to determine the capacity for adsorption of the given sorbent. A standard isothermal graph was plotted between equilibrium fluoride concentration in solution (C_e_) and fluoride sorbed onto sorbent at equilibrium (q_e_) for initial fluoride concentrations ranging from 5 to 70 mg/L. Figure 6, shows the standard isothermal plot for fluoride sorbed onto LIB and Fig. 7 shows the standard isotherm plot for adsorption of fluoride onto bauxite. Both the sorbents showed almost a similar trend of high fluoride uptake at lower concentrations and with progressive increase in concentration of fluoride, the rate of fluoride uptake gradually decreased probably due to exhaustion of active sorption sites on the sorbents. Results of the experimental data were modeled using Langmuir and Freundlich isothermal models, to arrive at the best fitting model.

#### Langmuir and freundlich isotherm models

Langmuir isotherm assumes monolayer coverage of adsorbate on sorbent. The linear form of Langmuir isotherm model is given in Eq. (4) (Langmuir 1916).

4 $$\left( {{\text{C}}_{\text{e}} / {\text{q}}_{\text{e}} } \right) = 1 / {\text{q}}_{\hbox{max} } \cdot {\text{b}} + {\text{C}}_{\text{e}} / {\text{q}}_{\hbox{max} }$$

where C_e_ (mg/L) is concentration of fluoride in solution at equilibrium, q_e_ (mg/g) is amount of fluoride sorbed onto the sorbent at equilibrium, q_max_ (mg/g) is maximum adsorption capacity and b (L/mg) is a constant related to energy.

Freundlich isotherm model is based on the assumption that the surface of the sorbent is heterogeneous, with different sorption sites possessing different energies of sorption (Freundlich 1906). The linear form of Freundlich equation is given by Eq. (5).

5 $$\log \left( {{\text{q}}_{\text{e}} } \right) = \log \,{\text{k}}_{\text{f}} + 1 / {\text{n}}\log {\text{C}}_{\text{e}}$$

where k_f_ represents relative adsorption capacity and n represents the intensity of sorption. The value of (1/n) suggests the nature of sorption. Value of 1/n greater than one suggests physisorption, whereas its value <1 suggests chemisorption (Freundlich 1906). Table 4 shows the values of all parameters of Freundlich model. It can be observed that the value of (1/n) is <1 suggesting chemisorption. From Table 4, it can be inferred that the best fitting isothermal model is Langmuir model, based on closeness of R^2^ value to 1. Also the predicted adsorption capacity of LIB is 18.18 mg/g, which is close to experimental value (17.2 mg/g). Similarly, in case of bauxite, the predicted and experimental values of maximum adsorption capacity are 7.72 and 3.2 mg/g respectively. The maximum adsorption capacities of various sorbents such as bauxite (Sujana and Anand 2011), lanthanum modified activated alumina (Cheng et al. 2014), lanthanum impregnated chitosan flakes (Jagtap et al. 2011), lanthanum incorporated chitosan beads (Bansiwal et al. 2009), metallurgical grade alumina (Pietrelli 2005),mixed rare earth oxides (Raichur and Basu 2001) and titanium rich bauxite (Das et al. 2005) along with the presently investigated sorbents are given in Table 5, for comparison. It can be observed from this table that the adsorption capacity of LIB is higher than all other sorbents except lanthanum hydroxide, which has an exceptionally high value of 242.2 mg/g. Similarly sorption capacity of bauxite used in this study has higher value of 7.7 mg/g compared to sorption capacity of bauxite obtained by Sujana and Anand (2011), probably due to calcination.

**Table 4:** Comparison of isothermal constants for adsorption of fluoride onto LIB and bauxite

	Isotherm model	Langmuir	Freundlich
LIB	Isotherm parameters	q_max_ = 18.18 mg/g b = 0.541 R^2^ = 0.997	K_f_ = 5.794 mg/g n = 2.753 R^2^ = 0.805
Bauxite	Isotherm parameters	q_max_ = 7.722 mg/g b = 0.379 R^2^ = 0.992	K_f_ = 1.902 mg/g n = 2.085 R^2^ = 0.861

**Table 5:** Comparison of isothermal constants for adsorption of fluoride onto LIB and bauxite

Adsorbent	q_max_(mg/g)	References
Bauxite	5.16	Sujana and Anand (2011)
Titanium rich bauxite	4.13	Das et al. (2005)
Lanthanum hydroxide	242.2	Na and Park (2010)
Lanthanum modified activated alumina	6.7	Cheng et al. (2014)
Lanthanum impregnated chitosan flakes	1.27	Jagtap et al. (2011)
Lanthanum incorporated chitosan beads	4.7	Bansiwal et al. (2009)
Alumina metallurgical grade	12.57	Pietrelli (2005)
Magnesia amended activated alumina	10.12	Maliyekkal et al. (2008)
Mixed rare earth oxides	12.5	Raichur and Basu (2001)
Lanthanum impregnated bauxite	18.18	Present study
Bauxite	7.722	Present study

### Influence of pH

**Fig. 8:**
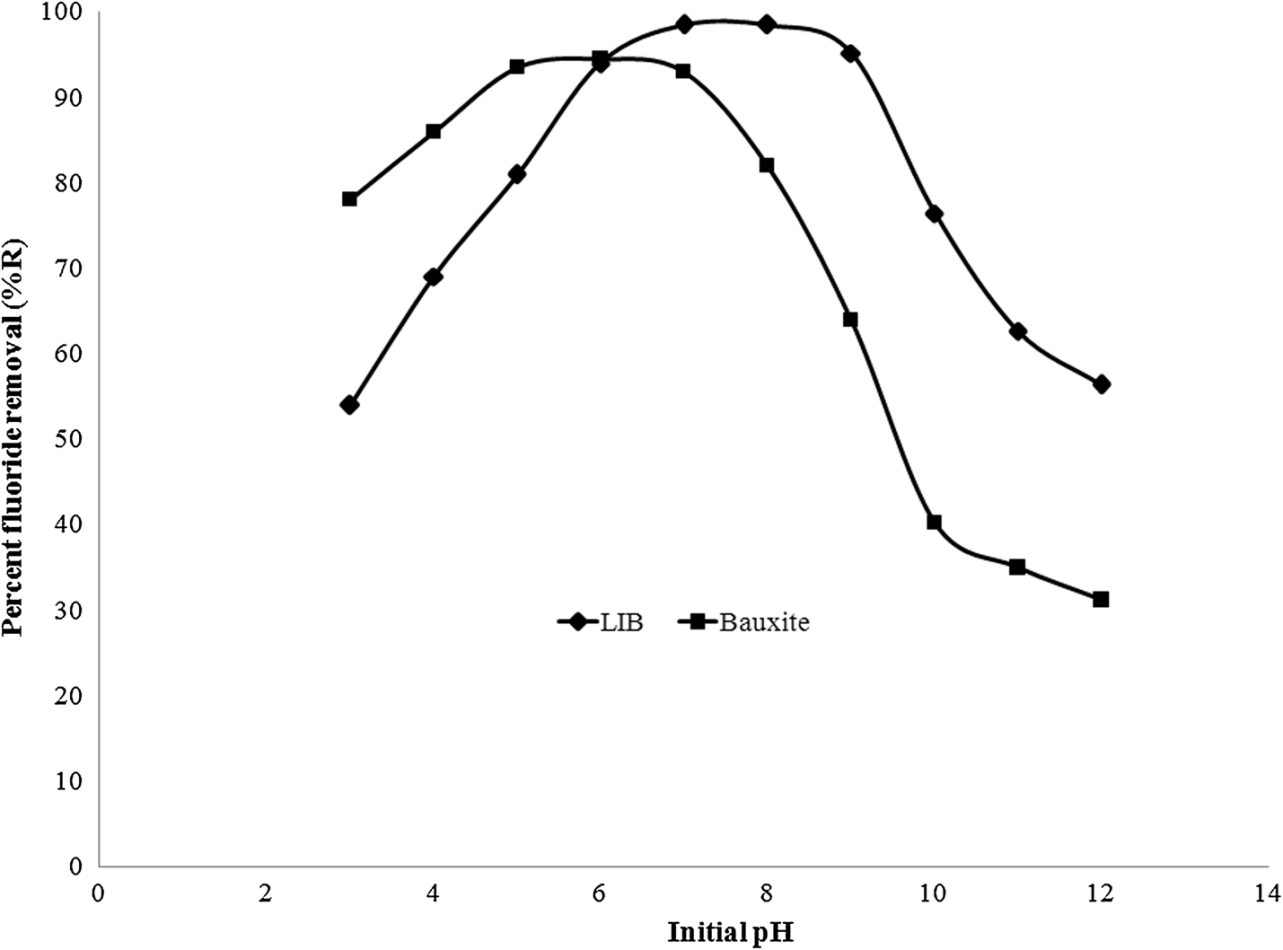
Influence of pH on defluoridation by LIB and bauxite

pH plays a significant role in adsorption. pH studies were conducted for removal of fluoride from water using LIB and bauxite by adjusting the starting pH of solution in the pH range 3–12 and the results are presented in Fig. 8. It can be observed that for LIB the optimum fluoride removal was from pH 6.5 to 8.5, whereas for bauxite the optimum removal of fluoride was between 5.0 and 6.5. Beyond this range either at lower or higher pH values fluoride removal was observed to be significantly less. This pH range coincides with the pH range observed for removal of fluoride using titanium rich bauxite (Pietrelli 2005). It was observed that the equilibrium pH of solution increased by about 0.2–0.4 U than the initially adjusted pH, for both sorbents after adsorption at pH < 8. From pH above 8.0 the pH drift was not observed. As the drift in pH was observed only in the optimal fluoride removal pH range for both sorbents, which is eventually the natural occurring pH of water, the impact of drift was neglected. At low pH values H^+^ ions predominate and so could form a bond with fluoride forming HF (Maliyekkal et al. 2008). pKa of HF is 3.16 (Shen and Schafer 2014). When pH value of solution is lesser than 3.16, weakly charged HF ions predominate, which may not get readily adsorbed onto sorbent. However, when the value of pH is increased higher than 3.16, F^−^ ions predominate, which get readily adsorbed. So, a gradual increase in removal of fluoride is observed after this pH value. Also, below pH value of 5, metallic salts present in bauxite, dissociate into their respective cations such as Al^3+^ and dissolve into solution (Adams 1999), thereby decreasing the active sorption sites on the surface of bauxite. So, removal of fluoride by bauxite is less below pH 5. Similarly below pH 7, lanthanum exists as La^3+^ ion (Cetiner and Xiong 2008), which dissociates itself from LIB causing a reduction in the active sites of sorption on LIB. However, when pH of solution containing bauxite is raised to more than 5 and that of LIB to more than 7, the cations such as Al^3+^ and La^3+^, get converted to their respective salts and stay on the sorbents, thereby aiding in adsorption. So, removal of fluoride, using bauxite increases from pH 5 onwards and removal of fluoride using LIB increases from pH 7 onwards. However further investigation is warranted to know the combined influence of dissolution of all the metal ions which influence sorption of fluoride at various pH values in lower pH range. At higher pH values OH^−^ ions predominate which compete for active sorption sites on the surface of sorbent with F^−^ ions. Thus due to the phenomena of competitive adsorption, sorption of fluoride ions onto the surface of sorbent could decrease. Also the pHzpc of LIB and bauxite were found to be 8.2 ± 0.1 and 6.0 ± 0.1 respectively. This further justifies the optimal sorption of fluoride in the observed ranges. The charge on the surface of a sorbent remains positive from lower values of pH to the value of pH_zpc_. From this point onwards any further increase in pH of solution changes the surface charge of sorbent from positive to negative.

### Influence of anions

**Fig. 9:**
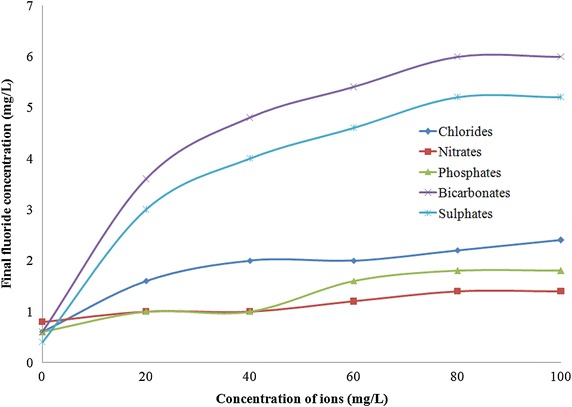
Influence of competing anions on sorption of fluoride onto LIB (Initial fluoride = 20 mg/L, Sorbent dose = 2 g/L)

Groundwater may consists of several anions such as Cl^−^, SO_4_^2−^, PO_4_^3−^, HCO^3−^ and NO^3−^ in addition to fluoride, which might compete with fluoride for sorption onto the active sites on the sorbent (Fink and Lindsay 1936). This might reduce the sorption of fluoride onto LIB. So, the above mentioned ions were added each individually in concentrations up to 100 mg/L along with fluoride of concentration 20 mg/L in distilled water and sorption experiments were conducted. The impact of the anions tested here on sorption of fluoride onto LIB is depicted in Fig. 9. It can be observed that except nitrates, addition of other ions increased the final fluoride concentrations after adsorption to more than 1.5 mg/L. As lanthanum is predominant in LIB, it could possibly have less affinity for nitrates compared to other ions.

### Regeneration studies

**Fig. 10:**
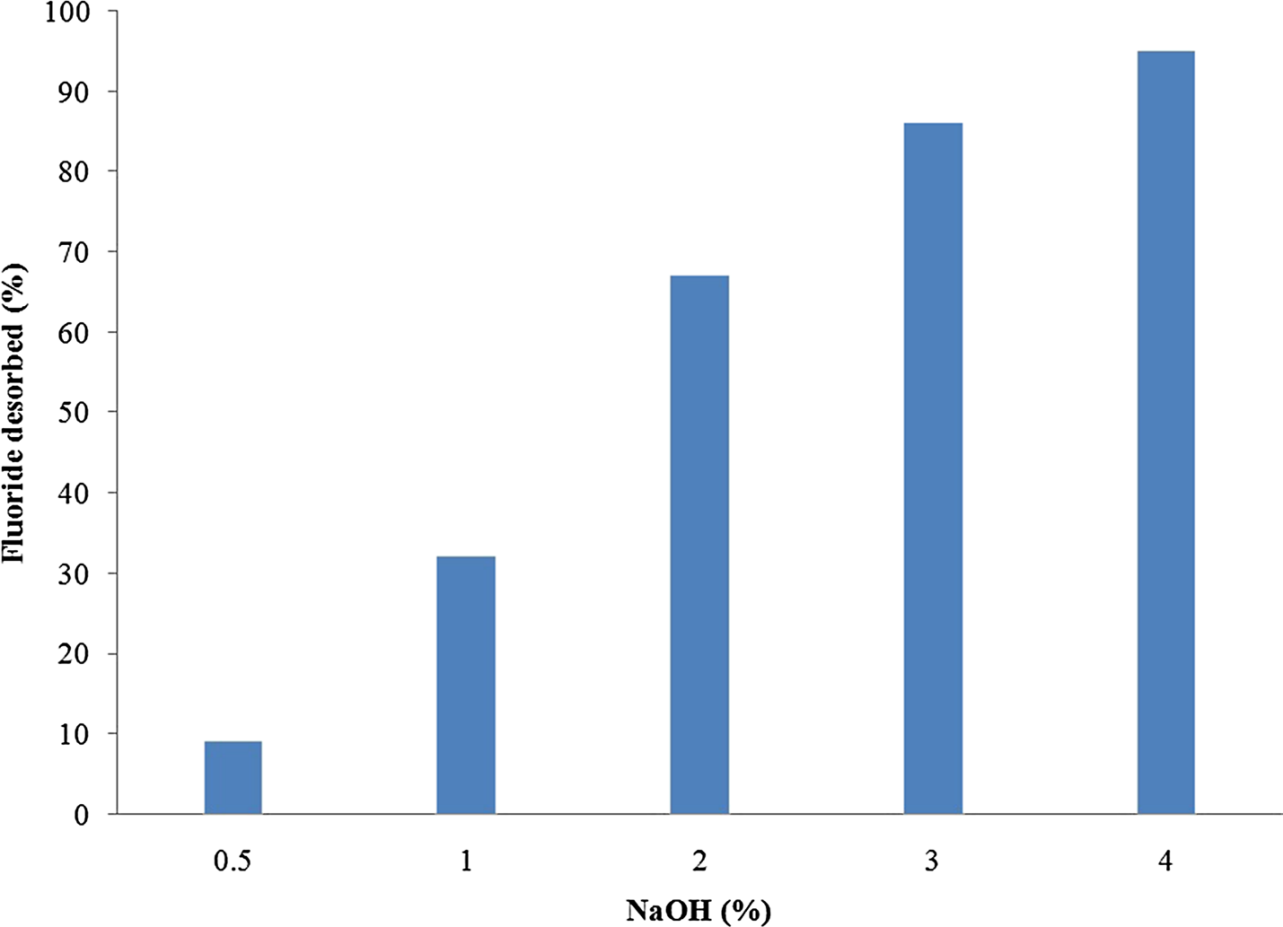
Effect of NaOH concentration on the fluoride desorption

To determine the potential of re-applicability of spent sorbent, regeneration studies were conducted. Initially adsorption experiments were conducted with 20 mg/L, aqueous fluoride solution using LIB, under optimum conditions. Loaded LIB was agitated with distilled water and the desorption was found to be very low (36 %). Therefore desorption studies were carried out using various eluents such as NaOH and H_2_SO_4_. It was observed that NaOH could elute fluoride from the loaded sorbent successfully. The influence of various concentrations of NaOH in eluting the sorbent loaded to capacity with LIB is presented in Fig. 10. A 4 % NaOH solution could elute nearly 95 % of fluoride. This could be due to exchange of OH ion in NaOH with F^−^ ion, as given in Eq. 6.

6 $${\text{MF}} + {\text{NaOH}} \to {\text{MOH}} + {\text{NaF}}$$

**Fig. 11:**
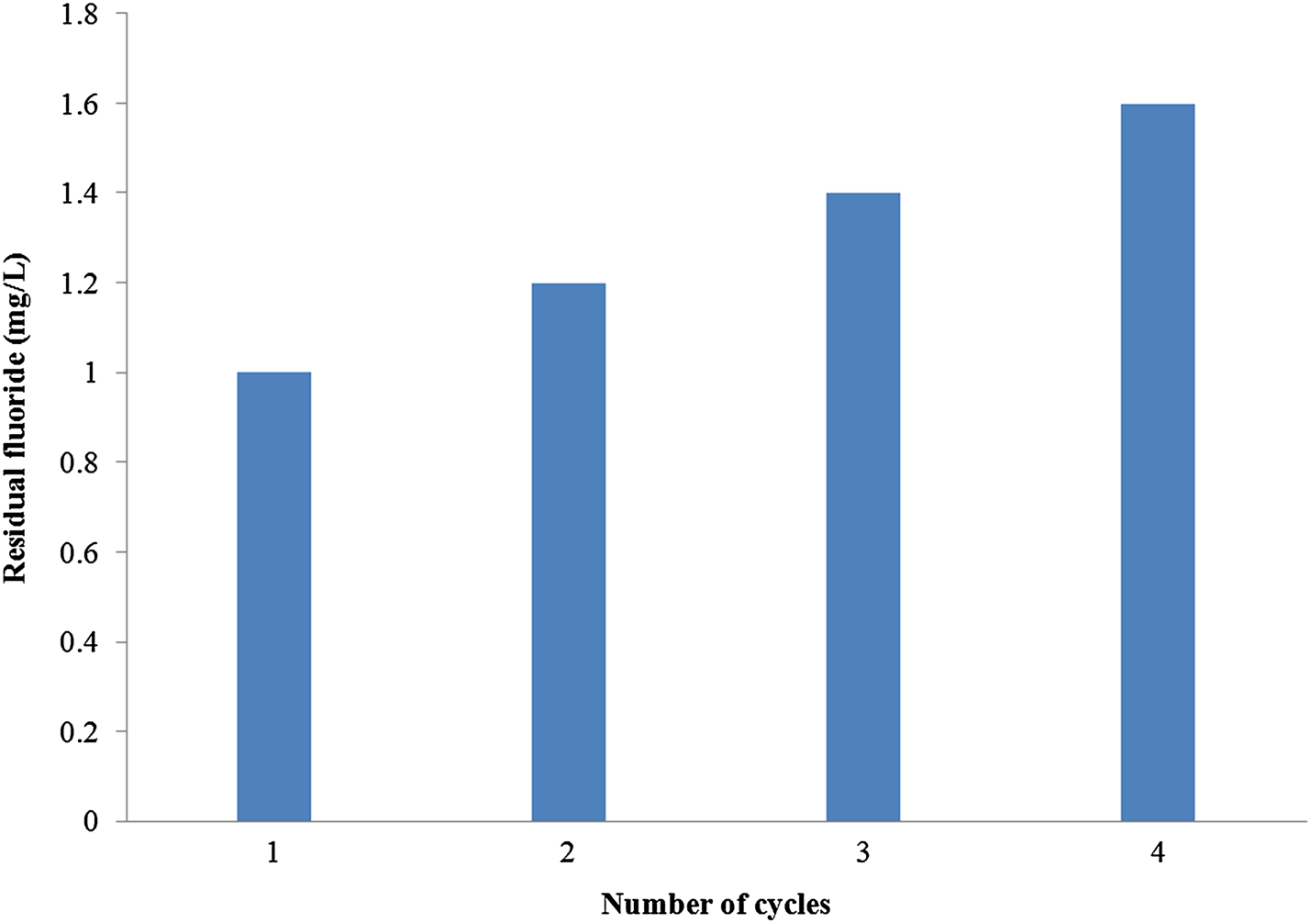
Number of cycles of defluoridation by LIB after regeneration

The regenerated sorbent thus obtained was again used as a sorbent, and subsequently eluted with NaOH. Such cycles were repeated and the maximum removal of fluoride after each cycle is presented in Fig. 11. It can be observed that as against predicted 0.7 mg/L of residual fluoride, the residual fluoride after first cycle was found to be 1 mg/L and it rose to 1.4 mg/L after 3 cycles. Further, after 4th cycle, the residual fluoride was found to be 1.6 mg/L which exceeds the permissible limit. So, 3 cycles of regeneration can be considered safe for LIB. However, this is not quite in agreement with 5 % of fluoride residing on sorbent after elution after each cycle. Further detailed experimentation is warranted to understand the exact reason underlying this. As only 90 % of fluoride is eluted after each cycle, the defluoridation capacity decreases after each cycle. Also with each cycle of elution with NaOH, OH ions accumulation on adsorbent increases which reduces its defluoridation capacity. It can be observed that the regenerated sorbent can be used successfully up to 4 cycles without significant loss, in the sorption potential.

## Conclusions

In this study a novel sorbent, LIB was prepared and investigated for removal of fluoride from water. LIB exhibited good sorption potential of 18.18 mg/g, compared to several other sorbents, collected from literature. However, further investigation is warranted to find the exact sorption capacity of LIB under natural groundwater conditions. The main conclusions are

- LIB was prepared by thermal impregnation method, and it reduced fluoride from distilled water from 20 to 0.7 mg/L.
- LIB at a dose of 2 g/L removed fluoride up to 99 % from an initial concentration of 20 mg/L of aqueous fluoride solution, whereas bauxite at a dose of 6 g/L removed up to 94 % of fluoride from an initial concentration of 20 mg/L of aqueous fluoride solution.
- The time taken for defluoridation by LIB was 120 min and it followed pseudo second order reaction, which indicates that the mechanism involved could be chemisorption. Pore diffusion seems to be the rate limiting step. The time taken for defluoridation by bauxite was 150 min.
- The sorption process by LIB conformed to Langmuir isotherm model. The maximum sorption capacity according to this model was 18.18 mg/g, which was close to observed experimental values. Bauxite followed a similar trend with a best fit to Langmuir isotherm model. The maximum sorption capacity of bauxite was found to be 7.722 mg/g.
- A pH range of 6.5–8.5 was found to be optimum for LIB, for removal of fluoride from water, which is a naturally occurring pH for waters. Bauxite exhibited optimum fluoride removal from pH 5.0 to 6.5.
- Addition of NO^3−^ to aqueous fluoride solution water brought the residual fluoride concentration after sorption to 1.4 mg/L, whereas other individual ions added such as Cl^−^, SO_4_^2−^, PO_4_^3−^ and HCO_3_^−^ caused the final fluoride concentration after sorption to be more than 1.5 mg/L, probably due to competition of ions.
- 4 % NaOH regenerated LIB by 95 % and the effective number of cycles after regenerations were found to be 3 for removal of fluoride up to permissible limit.
